# Application and research progress on artificial intelligence in the quality of Traditional Chinese Medicine

**DOI:** 10.3389/fphar.2025.1687681

**Published:** 2025-10-17

**Authors:** Mei-Yu Li, Jun-Qing Zhu, Xiao-Nan Liu, Meng-Yue Wu, Kun Dong, Xiao-Yan Li, Peng Gao, Zhi-Hui Jiang

**Affiliations:** Shandong Key Laboratory of Digital Traditional Chinese Medicine, Institute of Pharmacy (Institute of TCM Health Industrial Technology), Shandong University of Traditional Chinese Medicine, Jinan, China

**Keywords:** artificial intelligence, bioinformatics, quality control, traditional Chinese Medicine, mechanism, multi-omics technologies

## Abstract

The clinical safety and therapeutic performance of Traditional Chinese Medicine (TCM) are closely tied to its quality. However, with the rapid expansion of the TCM industry, conventional quality control approaches based on empirical observations and single-metabolite quantification have become increasingly inadequate for addressing the complex and variable requirements of quality assessment. In recent years, artificial intelligence (AI)—with strong capabilities in data processing and pattern recognition—has emerged as a promising tool for establishing predictive models to efficiently handle heterogeneous, multi-source datasets (such as spectra, chromatograms, images, and textual information). This enables intelligent prediction of quality indicators and anomaly detection, and offering novel strategies for modernizing TCM quality control. This review provides a comprehensive synthesis of commonly applied machine learning and deep learning algorithms, systematically outlining recent advances in AI-enabled sensing applications such as image recognition, odor analysis, authenticity verification, origin tracing, quality grading, and storage-age determination. It further emphasizes the integration of AI with multi-omics and bioinformatics approaches for efficacy-oriented evaluation and safety assessment, including identification of Q-markers, elucidation of pharmacodynamic mechanisms, and predictive modeling of both endogenous and exogenous toxic metabolites. It also identifies key challenges and technical bottlenecks, and outlines priorities for building scalable, regulation-aware, data-driven quality-control systems that support the sustainable, high-quality development of the TCM industry.

## 1 Introduction

Traditional Chinese Medicine (TCM) receives growing international attention for its integrative treatment principles, clinically validated efficacy, favorable safety profile, and emphasis on personalized care (Monakhova et al., 2018). TCM has long emphasized the integration of medicinal materials with clinical practice. In this context, identifying, selecting, and applying TCM are closely tied to clinical decisions. Among these factors, the consistency of TCM quality is essential for maintaining the clinical reliability of TCM interventions (Zhao J. et al., 2018; Liu C.-L. et al., 2024; Soltani et al., 2025).

However, the heterogeneous sources of medicinal materials, chemical complexity of TCM, absence of unified quality control standards, and incomplete understanding of pharmacological mechanisms collectively lead to substantial inconsistencies in the quality and therapeutic performance of TCM (Wang and Li, 2022; Busia, 2024). Conventional quality control in TCM primarily relies on sensory-based techniques and basic physicochemical assessments. These include traditional diagnostic methods such as *wang*, *wen*, *wen*, and *qie* (inspection, olfaction and auscultation, inquiry, and palpation), as well as organoleptic evaluation to determine the Four Qi (cold, hot, warm, cool) and Five Flavors (pungent, sweet, sour, bitter, salty). Despite their convenience, such methods are inherently subjective and lack reproducibility, rendering them inadequate for modern quality evaluation standards (Li et al., 2020; Luo et al., 2024). Modern analytical technologies, including chromatography and spectroscopy, have enabled metabolite profiling systems. However, these platforms predominantly quantify selected marker metabolites. Such approaches often fail to capture the intrinsic complexity of TCM, which involves multiple bioactive metabolites acting synergistically through diverse targets and pathways. Furthermore, the correlation between such analytical data and actual pharmacological efficacy or safety remains weak (Li and Zhang, 2013). The widespread application of modern quality control approaches is also limited by their reliance on elaborate sample preparation, costly instrumentation, and the need for specialized personnel (Liu Y. et al., 2025).

The modernization and globalization of TCM demand the development of a standardized, data-driven, and intelligent quality control system to support accurate assessment and effective regulation of product quality. Advances in artificial intelligence (AI) technologies have opened new avenues for innovation in TCM quality control. Machine learning (ML) algorithms have been effectively deployed in phenotypic tasks such as origin traceability, species authentication, and quality grade classification, thereby improving the objectivity and consistency of TCM characterization. Meanwhile, the integration of AI with analytical platforms such as chromatography, mass spectrometry, and multi-omics technologies has advanced the automation and standardization of TCM quality evaluation (Caratti et al., 2024; Chi et al., 2024).

TCM-derived metabolites exhibit pronounced structural heterogeneity, while their pharmacological actions are often complex and synergistic. As a result, phenotypic-level intelligent recognition alone is insufficient to capture the multi-metabolite, multi-target and multi-pathway characteristics of TCM. In contrast, bioinformatics and multi-omics platforms—including transcriptomics, metabolomics, and proteomics—offer a multidimensional perspective on the underlying therapeutic mechanisms. When combined with AI-driven computational modeling, these approaches make it possible to trace the pathway from bioactive metabolites to defined molecular targets, and further to the modulation of signaling networks that mediate therapeutic efficacy (Soon et al., 2013; Liu and Guo, 2020; Wang et al., 2021; Li D. et al., 2022).

Within the intelligent quality control framework, AI-assisted phenotypic recognition—covering morphological features and spectral fingerprints—functions as the primary entry point for assessment. At a deeper analytical tier, the integration of AI with bioinformatics enables the elucidation of intricate target–pathway–outcome relationships, thereby linking chemical composition and pharmacodynamic mechanisms to the holistic efficacy of TCM. Notably, such a dual-level strategy strengthens the scientific rigor and reproducibility of quality evaluation. Collectively, these advances pave the way for the establishment of an efficacy-driven, mechanism-informed quality control system.

## 2 Artificial intelligence technologies

**FIGURE 1:**
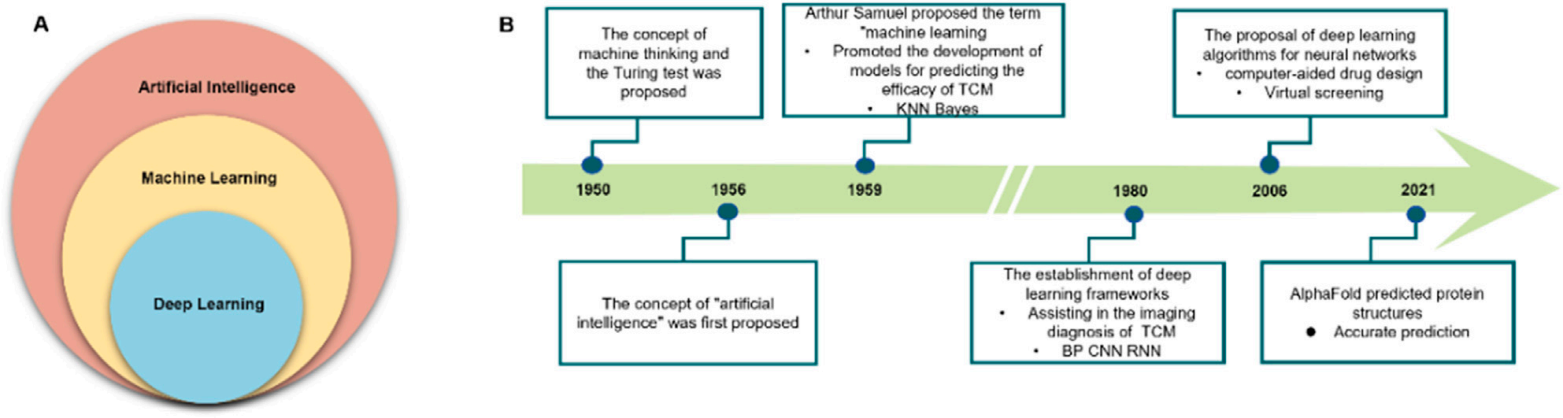
Artificial intelligence (AI), machine learning (ML), and deep learning (DL): advances in healthcare. **(A)** Relationship among AI, ML, and DL. **(B)** Evolution of AI in daily life and healthcare.

Among various AI approaches, ML—particularly its subset deep learning (DL)—has found the broadest application in the medical field (Russell and Norvig, 2022) (Figure 1A). Since its inception, AI has maintained a close connection with healthcare and has gradually expanded to encompass diverse domains of human activity (Figure 1B) (Aydın Temel et al., 2023). In the context of TCM research, AI facilitates the recognition, modeling, and prediction of complex and heterogeneous data. It enables automated extraction of sensory features and chemical fingerprints—parameters that have traditionally been challenging to quantify—and can be integrated with mechanistic models to inform efficacy and safety assessment in TCM.

### 2.1 Machine learning techniques

**FIGURE 2:**
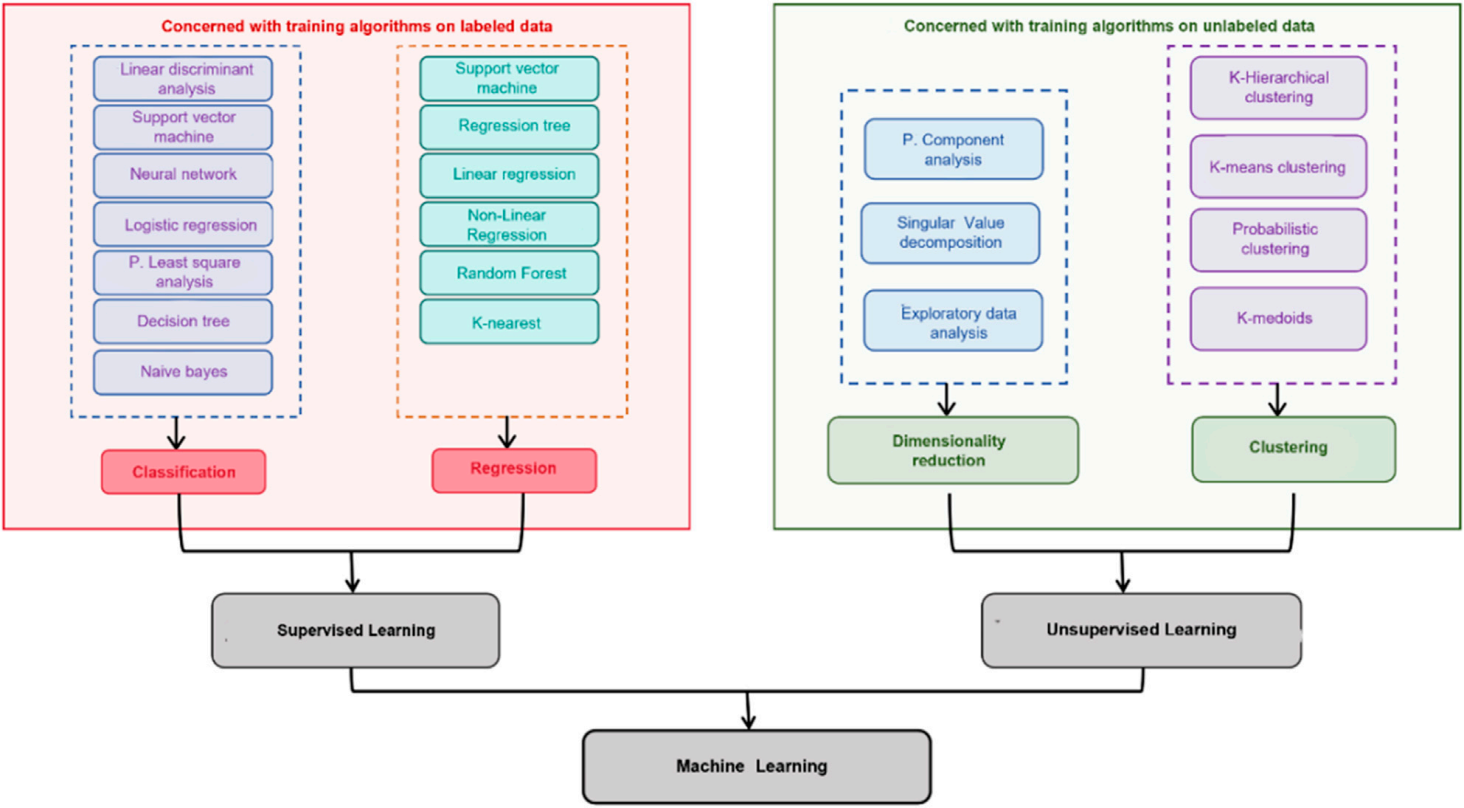
Classification of ML techniques: distinction between supervised learning and unsupervised learning.

ML is typically categorized into three main types: supervised learning, unsupervised learning, and reinforcement learning. This review primarily focuses on supervised learning and unsupervised learning relevant to TCM quality assessment (Figure 2).

#### 2.1.1 Supervised learning

Supervised learning constitutes a fundamental framework within ML, wherein models are trained on labeled datasets to establish explicit mappings between inputs and outputs (Suriyaamporn et al., 2024). By capturing such relationships, supervised algorithms can perform both classification tasks and regression tasks with high predictive accuracy, where classification assigns inputs to discrete categories, while regression predicts continuous outcomes (Maione et al., 2019). A broad spectrum of algorithms is routinely employed, including Naïve Bayes (NB), linear discriminant analysis (LDA), decision trees (DT), random forests (RF), support vector machines (SVM), logistic regression (LR), k-nearest neighbors (KNN), and regression approaches such as simple linear, multiple linear, and polynomial regression. These models support both classification and regression pipelines commonly encountered in TCM quality evaluation, spectrum–structure correlation, and pharmacokinetic modeling.

NB: NB is a probabilistic classifier grounded in Bayes’ theorem and the conditional-independence assumption among features (Ou et al., 2025). By combining prior distributions with class-conditional likelihoods, it estimates posterior probabilities and assigns each sample to the most probable class. Despite its austerity, NB is computationally frugal, robust on small or high-dimensional spaces, and comparatively tolerant of irrelevant variables. Notably, NB has been used across TCM-related tasks—including classical medical text categorization, adulteration screening of decoction pieces, and time-series analyses of pharmacodynamic readouts—where rapid, baseline performance is desirable.

LDA: LDA is a supervised method for classification and dimensionality reduction that seeks projections maximizing interclass separation while minimizing intraclass variance (Lam et al., 2024). Assuming multivariate normality with a common covariance structure, LDA yields linear decision boundaries, which in turn facilitate interpretability and efficient computation. In contrast to previous reports focusing solely on visualization, recent TCM studies deploy LDA for spectral feature extraction (e.g., NIR, HSI), geographic-origin authentication, and prediction of chemical-composition profiles aligned with Q-markers frameworks and multi-omics fingerprints.

DT: DT is supervised learners applicable to both classification and regression, representing decision rules in a hierarchical, tree-like structure. Through recursive partitioning on feature thresholds, DTs generate models that are straightforward to visualize and implement (Sarker, 2021). In TCM research, they have been widely adopted for efficacy prediction, quantification of active metabolites, and routine quality control of medicinal materials (Ren et al., 2022; Zhang W. et al., 2025).

**FIGURE 3:**
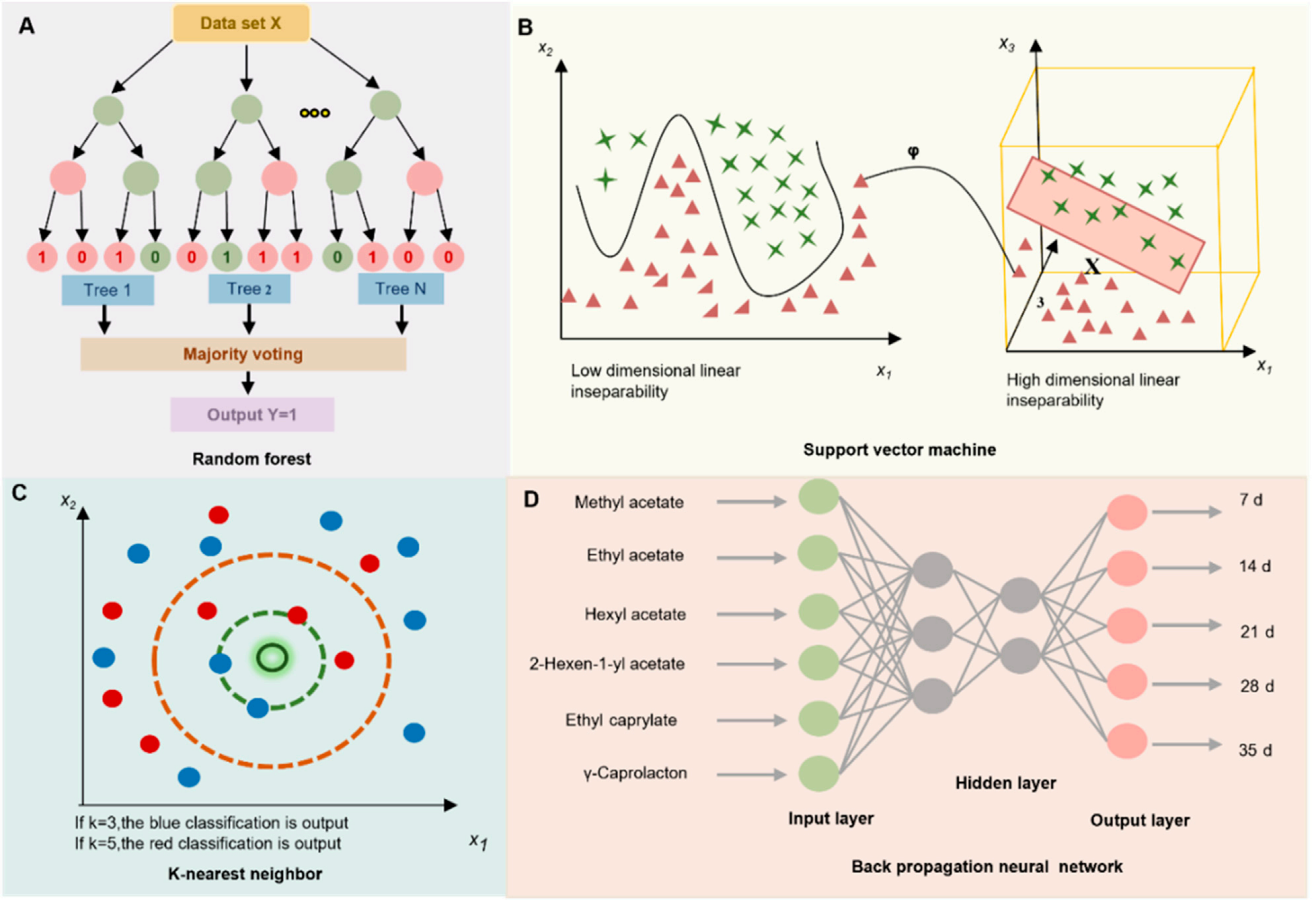
Schematic representation of 4 ML models employed in TCM research: Random Forest (RF), Support Vector Machine (SVM), K-Nearest Neighbors (KNN), and Backpropagation Neural Network (BPNN).

RF: RF is an ensemble learning approach that builds multiple decision trees and aggregates their outputs to generate the final prediction. In classification tasks, the predicted class is determined by majority voting, whereas in regression tasks, the results are obtained by averaging the outputs of individual tree (Figure 3A). Due to its strong robustness to noise and ability to capture complex feature interactions, RF has shown high accuracy for botanical drug origin traceability and in recognizing multi-metabolite feature patterns characteristic of TCM formulations (Gong et al., 2023).

SVM: SVM is a versatile ML technique used for various purposes, including classification and regression. It projects input data into a high-dimensional feature space using kernel functions, enabling the linear separation of inherently nonlinear patterns (Figure 3B) (Kremer et al., 2014). In the TCM domain, SVM has been widely employed for spectral data analysis—such as near-infrared spectroscopy (NIR) and hyperspectral imaging (HSI)—as well as for classifying pharmacological effects across different decoction pieces and TCM prescriptions (Kumari et al., 2021; Ouyang et al., 2023).

LR: LR is a supervised statistical learning method used chiefly for binary classification and, via standard extensions, multiclass tasks. It maps a linear combination of predictors through the logistic (sigmoid) link to obtain class-membership probabilities, with coefficients directly interpretable as odds ratios. In TCM research, LR has been applied to classify TCM species from chromatographic or spectral fingerprints, to predict the therapeutic efficacy of metabolites, and to assess toxicity risks in complex multi-metabolite formulations, consistent with the field’s focus on holistic efficacy and rigorous quality evaluation.

KNN: K-NN is an ML algorithm for classification and regression. It assigns a label to a sample based on the majority class among its k nearest neighbors, identified using distance metrics such as Euclidean or Mahalanobis distance (Figure 3C). Owing to its ease of implementation and dependable performance, this approach has proven to be a practical choice for a range of applications in TCM, including species authentication of TCM materials, tracing geographic origin, and assigning quality grades (Boniecki et al., 2014; Li et al., 2022b).

BPNN: A feed-forward artificial neural network trained using the backpropagation learning algorithm, the BPNN is particularly effective in modeling complex and nonlinear relationships between chemical composition and pharmacological efficacy (Figure 3D). In the field of TCM research, BPNN has been extensively applied to optimize processing parameters, enhance extraction protocols, and predict multi-index quality attributes (Yi, 2019).

Regression analysis—an essential supervised approach for continuous outcomes—has been increasingly applied in TCM to quantify links between chemical composition and quality attributes. By integrating spectral or metabolomic data with regression models such as partial least squares (PLS), these workflows enable rapid estimation of active metabolite levels, discrimination of authentic metabolites from adulterants, and the construction of robust quality-control frameworks (Moreno-Torres et al., 2024; Zhang X. et al., 2024).

#### 2.1.2 Unsupervised learning

Unsupervised learning refers to algorithms that discover hidden structures and patterns in unlabeled data without predefined outputs. Typical methods include clustering and dimensionality reduction (Castiglioni et al., 2021).The following introduces several common unsupervised algorithms, including K-means clustering (K-means), Density-Based Spatial Clustering of Applications with Noise (DBSCAN), Gaussian Mixture Models (GMMs) and Principal Metabolite Analysis (PCA).

K-means: K-means is an unsupervised learning algorithm that partitions data into *k* clusters by minimizing the within-cluster variance. It iteratively assigns samples to the nearest cluster centroid and updates centroids until convergence.

DBSCAN:DBSCAN is an unsupervised clustering algorithm that groups data points based on density, identifying high-density regions as clusters and treating sparse points as noise or outliers. Unlike K-means, it does not require specifying the number of clusters in advance and can discover clusters of arbitrary shape.

GMMs: GMMs are probabilistic unsupervised learning algorithms that assume data are generated from a mixture of multiple Gaussian distributions, each representing a cluster. Unlike K-means, GMMs provide soft clustering by assigning probabilities for each data point belonging to different clusters. In TCM research, GMMs have been used to classify TCM samples based on spectral or metabolomic data and to distinguish authentic materials from adulterants with overlapping chemical features (Bai and Zhang, 2024).

PCA: A linear dimensionality reduction method that transforms correlated variables into a smaller set of uncorrelated principal metabolites while retaining most of the variance. In TCM, PCA is widely used to simplify spectral or chromatographic data for quality evaluation (Thapa et al., 2025).

In parallel, unsupervised techniques—such as hierarchical clustering, t-SNE, and autoencoders—have been applied to data visualization, authenticity verification of decoction pieces, adulteration detection, and quality evaluation (Paolanti and Frontoni, 2020; Bansal et al., 2024). Taken together, supervised and unsupervised workflows constitute an integrated ML toolkit that supports classification, regression, clustering, and dimensionality reduction across complex TCM systems.

### 2.2 Deep learning techniques

DL, a prominent branch of supervised learning within ML, is known for its remarkable capacity to extract high-level, discriminative features from complex datasets. Leveraging neural network (NN) architectures, DL enables data-driven decision-making in intricate systems by uncovering latent patterns from both large-scale structured data—such as images, spectral profiles, and textual corpora—and diverse unstructured datasets (Chen et al., 2022). Its superior representational and predictive capacity has positioned DL as a pivotal methodological tool in data-intensive TCM research.

Convolutional Neural Network (CNN): CNNs typically comprise convolutional layers, pooling layers, and fully connected layers. Through convolutional operations, they automatically learn hierarchical feature representations, which makes them particularly effective for high-dimensional image and signal processing in big data environments (Zeng et al., 2023a). Within TCM, CNNs have been applied to analyze surface morphology and microstructural features of medicinal materials, facilitating image-based authentication, geographic origin tracing, and the detection of pest (Kabir et al., 2022a).

Recurrent Neural Network (RNN): RNNs are designed to capture temporal dependencies and sequential patterns, rendering them well suited for time series and textual data. They have achieved wide application in natural language processing and biomedical literature mining. In the TCM context, RNNs support structured knowledge extraction from classical medical texts, the modeling of prescription sequences, and the analysis of biological time-series data, thereby contributing to knowledge standardization and semantic modeling (Tian et al., 2024).

Deep Neural Network (DNN): DNNs, characterized by multiple hidden layers, can model complex, high-dimensional relationships between input features and target outputs. In TCM research, they have been used to develop predictive models of pharmacological efficacy based on chemical composition profiles. Such end-to-end learning frameworks allow direct inference of pharmacological mechanisms from raw, multi-modal data (He et al., 2015).

Beyond CNN, RNN, and DNN, DL encompasses other influential architectures. Generative Adversarial Networks (GANs) can generate realistic synthetic data to augment limited datasets; Variational Autoencoders (VAEs) enable efficient feature compression and latent space modeling; and Deep Reinforcement Learning (DRL) optimizes decision-making policies through interaction with complex environments. These methods are widely adopted in fields such as natural language processing, robotics, and autonomous systems.

## 3 Mechanism-oriented transformation of TCM quality control: from sensing to mechanistic evaluation

**FIGURE 4:**
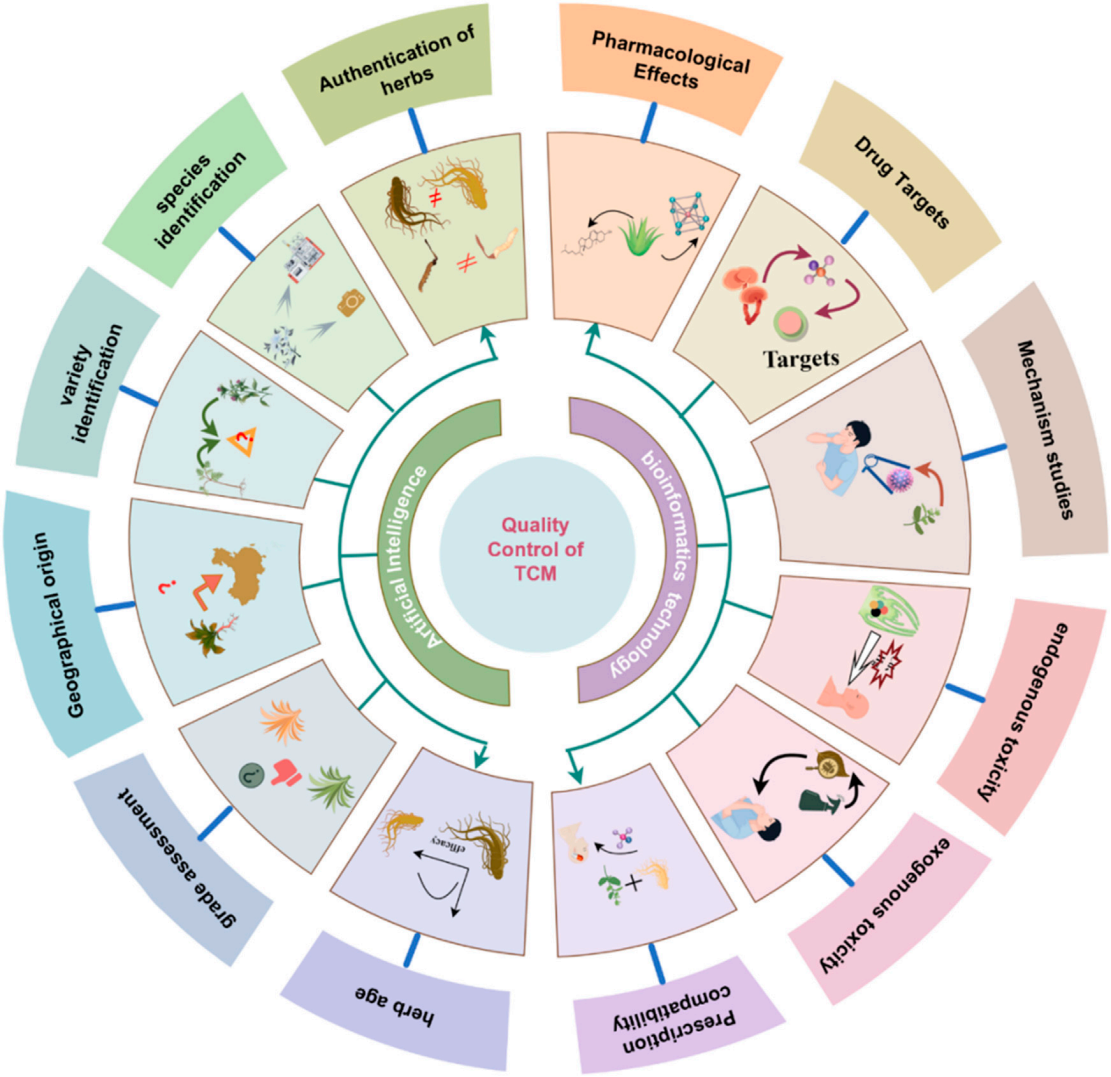
Applications of AI and bioinformatics in the quality control of TCM.

In recent years, the TCM industry have been undergoing a transition toward the integration of intelligent technologies. This shift reflects a broader transformation from experience- and perception-based evaluation to data-driven, mechanistically informed quality control model. AI has shown particular strengths in sensing. When integrated with bioinformatics, it supports the systematic construction of mechanistic frameworks for assessing efficacy and safety of TCM (Figure 4). This chapter focuses on the two major stages of this intelligent transformation and illustrates representative applications and recent advancements in TCM quality control.

### 3.1 AI-enabled sensing for the quality assessment of botanical drugs

**FIGURE 5:**
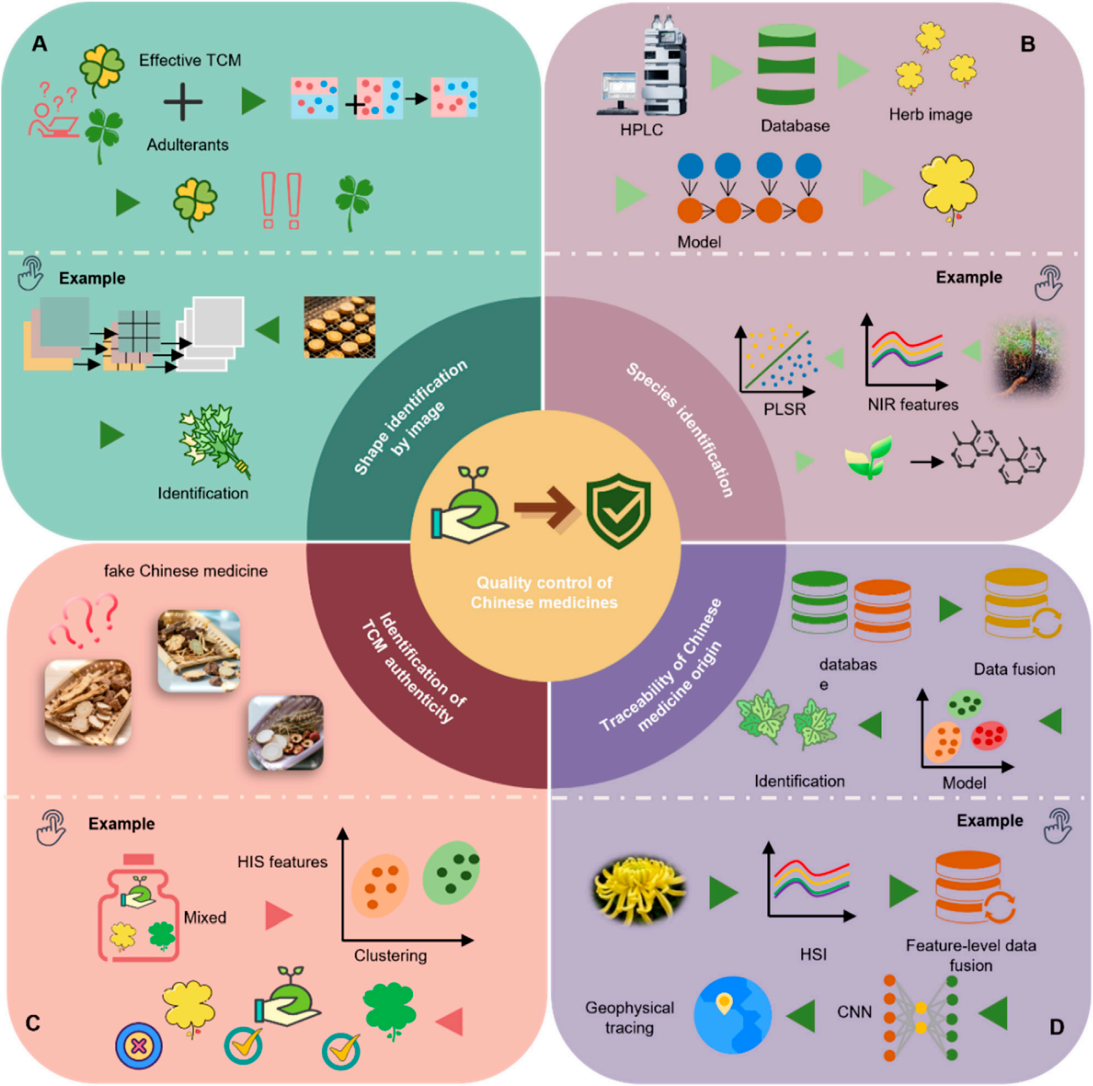
Representative applications of ML in TCM quality control. **(A)** Morphological identification based on image analysis: for instance, an improved ConvNeXt model is used for feature extraction and image classification to accurately identify TCM. **(B)** Species identification: for example, ATR-FTIR spectroscopy combined with partial least squares discriminant analysis (PLS-DA) is used to distinguish different varieties of *Ophiocordyceps sinensis* (Berk.) G.H. Sung, J.M. Sung, Hywel-Jones and Spatafora. **(C)** Authenticity verification: for example, hyperspectral imaging (HSI) combined with ML algorithms is used to differentiate naturally sun-dried from sulfur-fumigated TCM materials. **(D)** Origin tracing: for example, HSI combined with CNNs is applied to determine the production region of *Chrysanthemum morifolium* Ramat.

AI leverages a range of ML techniques to strengthen TCM quality control and address the inherent limitations of conventional multivariate statistical approaches. These technologies are increasingly utilized in key domains—including origin traceability, species authentication, and quality grading—reflecting the multi-dimensional nature of TCM quality evaluation (Figure 5). Table 1 summarizes representative case of AI applications in TCM quality control, highlighting algorithm types, data modalities, and specific application scenarios.

**TABLE 1:** Representative studies on the application of artificial intelligence in the quality control of TCM.

Main research contents	Research objective	Substance types	Algorithms	References
Image Recognition	Development of a smartphone-based TCM image recognition application	Smartphone	DNN	Sun et al. (2022b)
Image Recognition	Detection of various *Dendrobium officinale Kimura et Migo* via images	Smartphone	Improved YOLOv5; CV; ML	Chang et al. (2024)
Odor identification	Analysis of flavor metabolites in Boswellia carterii Birdw	HS-SPME-GC-MS; E-nose	PCA; PLS-DA	Chen et al. (2024c)
Odor identification	Rapid discrimination of tea quality grades based on odor	NIRS; E-nose	SVM; KNN; ANN	Xia et al. (2024)
Odor identification	Discrimination of cultivation methods based on the odor and taste of *Citrus reticulata* Blanco	GC-MS; HPLC-Q-TOF-MS; Electronic Nose; ET	PCA; PLS-DA	Li et al. (2022b)
Odor identification	Establishment of rice wine quality evaluation model	Flash GC Electronic Nose; NIR	OPLS-DA, DA, ELM, SVM, KNN, and LSTM; NN	Zhang et al. (2024d)
TCM authentication	Detection of adulteration in *Ziziphus jujuba* Mill. var. *spinosa* (Bunge) Hu ex H. F. Chou and improvement of prediction accuracy	FT-NIR Spectral Data	PLSR; SVM; KNN; ANN	Li et al. (2023)
TCM authentication	Identification and quantification of chemical dyes in Chinese medicinal products	SERS	PLSR; SVM; SSA-BP	Zhang et al. (2023a)
TCM authentication	Low-cost and rapid identification of *Crocus sativus* L. adulteration	FT-NIR	RF, SVM and CNN	Husaini et al. (2022)
TCM authentication	Discrimination of sun-dried vs sulfur-fumigated TCM products	HSI	PCA; PLS-DA	Zhang et al. (2016)
TCM authentication	Detection of adulteration and grade differentiation in Panax notoginseng (Burk.) F.H.Chen powder	ET, E-nose, and electronic eye-based AI-sensing Technologies	AIS; MIF	Li et al. (2024a)
Classification of TCM varieties	Evaluation of HSI combined with CNN for Fritillaria spp. identification	High-Spectral-Imaging HSI	SVM, PLS-DA, CNN	Kabir et al. (2022b)
Classification of TCM varieties	Discrimination of 10 different species of Ophiocordyceps sinensis (Berk.) G.H.Sung, J.M.Sung, Hywel-Jones and Spatafora	ATR-FTIR	PLS-DA; DD-SIMCA model	Li et al. (2022d)
Classification of TCM varieties	Species identification of Zanthoxyli Pericarpium using multiple analytical methods	Camera, Smartphone	CNN; SVM; BP	Tan et al. (2024)
Identification of origin	Development of a DL model for identifying the geographical origin and variety of Chrysanthemum morifolium Ramat	HSI	CNN; ML	Cai et al. (2023)
Identification of origin	Validation of NIR-HSI combined with ML for geographical origin detection of Lilium brownii F. E. Brown var. viridulum Baker	HSI	DT; LDA; KNN; SVM	Zhao et al. (2024)
Identification of origin	Identification of the geographical origin of Paeonia Paeonia lactiflora Pall	HSI	CNN; AM; KNN; RF; SVM	Cai (2023)
Identification of origin	Determination of geographical origin of Tetrastigma hemsleyanum Diels et Gilg	E-nose	PCA; PLS; HCA; Radial Basis Function (RBF)	Wu et al. (2022)
Grade evaluation	Development of a classification model to identify cultivation region, growth mode, species, and grade of *Astragalus membranaceus* (Fisch.) Bge	UPLC-MS/MS	Naive Bayes, KNN, RF, and four other ML algorithms	Wu et al. (2024)
Grade evaluation	Grade classification of TCM samples from different sources	UPLC	SVM	Li et al. (2022c)
Identification of storage age	Non-destructive method for accurate storage period identification of moxa wool	HSI	PCA; ELM; SVM; RF	Hu et al. (2024)
Identification of storage age	Analysis of storage duration of *Lonicera japonica* Thunb. across different months	E-Nose	LDA; NN	Xiong et al. (2014)

#### 3.1.1 Image recognition

Morphological identification is a fundamental step in ensuring the quality consistency of TCM. It requires systematic recognition of key features such as color, shape, leaf structure, and texture. However, inter-species similarity and intra-species morphological variation often compromise the reliability of manual identification, introducing subjectivity and reducing reproducibility.

AI-powered image recognition has emerged as a highly effective strategy for enhancing both the accuracy and efficiency of morphological identification in medicinal botanical drugs (Sun et al., 2022c). To overcome performance constraints, researchers have optimized ML architectures to improve classification precision and generalizability in botanical drug image recognition tasks (Xu et al., 2016). For example, transfer learning strategies have been applied using five pre-trained deep neural network architectures—ResNet34, DenseNet121, VGG11, ConvNeXt, and Swin Transformer—to improve classification accuracy. Among these, ConvNeXt achieved accuracies of 92.8% for Vietnamese samples and 92.5% for Indonesian samples, highlighting its strong adaptability to geographically diverse TCM datasets. In a related development, Xu et al. (2021) proposed an Attention Pyramid Network (APN) designed to dynamically capture multi-scale features from medicinal botanical drug images. Comparative evaluations revealed that APN consistently outperformed conventional Feature Pyramid Networks (FPN) within attention-based frameworks, confirming its superior accuracy and practical applicability.

The morphological authentication of certain medicinal species—*Dendrobium officinale* Kimura et Migo, for instance—has traditionally depended on expert judgment. Although highly effective when performed by trained professionals, such reliance poses practical challenges for non-specialist users, especially in cases involving subtle interspecific variations or closely related adulterants. To overcome the barrier and facilitate field deployment, researchers have developed an image-based recognition system optimized through an enhanced YOLOv5 algorithm. This system can be deployed on smartphones, enabling real-time identification of *D. officinale* Kimura et Migo and supporting on-site market regulation (Chang et al., 2024). Additionally, Sun et al. (2022b) design a DL–driven mobile application that maintains reliable performance on smartphones, thereby extending the accessibility of botanical drug recognition technologies to resource-limited settings.

#### 3.1.2 Odor identification

The identification and quality evaluation of TCM have historically relied on sensory assessment, focusing primarily on visual appearance, odor, and taste (Li et al., 2013). The therapeutic efficacy of TCM is often attributed to the synergistic interplay among multiple bioactive metabolites, some of which possess distinctive volatile profiles that contribute both to sensory recognition and to potential pharmacological effects (Ye et al., 2011). Consequently, reliable odor detection constitutes a critical foundation for the standardization and quality control of TCM. Conventional odor analysis—whether based on manual assessment, instrumental detection, or a combination of both—remains susceptible to variability introduced by individual factors (e.g., physical condition, mood) and environmental influences (e.g., temperature, humidity).

In recent years, ML models have gained notable traction in odor profiling and predictive analytics within TCM research, offering greater efficiency, reproducibility, and resistance to subjective bias than conventional sensory evaluation methods (Zeng et al., 2023b). One particularly effective strategy integrates headspace solid-phase microextraction gas chromatography–mass spectrometry (HS-SPME-GC-MS) with electronic nose (E-nose) systems, enabling comprehensive characterization of volatile organic metabolites (VOCs) and decoding complex aromatic signatures (Xia et al., 2024; Xu et al., 2024). For example, the aroma profile of *Boswellia carterii* Birdw. has long been regarded as a critical determinant of both product quality and consumer acceptance (Di Stefano et al., 2020). Using the dual analytical approach, researchers determined that alcohols constituted the dominant VOC class (22.15%), with p-cymenol identified as a principal contributor to the characteristic fragrance, thereby providing a chemical basis for quality differentiation (Chen X. et al., 2024). In a related application, Xia et al. (2024) employed the same methodology to differentiate tea grades according to olfactory profiles, highlighting its potential for sensory-driven quality assessment in TCM contexts.

Li et al. (2022b) combined E-nose and electronic tongue (ET) technologies with chemometric analysis to discriminate *Citrus reticulata* Blanco samples derived from different cultivation practices. Key volatile metabolites—including β-myrcene, limonene, β-trans-ocimene, γ-terpinene, and terpinolene—were identified as flavor-dominant metabolites and proposed as potential chemical markers for quality stratification. Huangjiu, a traditional fermented product frequently used as an excipient in TCM, can influence both physicochemical attributes and sensory characteristics of formulations, thereby modulating therapeutic outcomes (Wu et al., 2018). Taking Jimo rice wine (JRW) as a case study, researchers combined Flash gas chromatography–based electronic nose (Flash GC E-nose) and NIR with ML algorithms to develop a rapid quality evaluation model. This integrative strategy provides a reference framework for the intelligent quality control of other TCM and auxiliary materials (Zhang Z.-T. et al., 2024).

#### 3.1.3 TCM authentication

The presence of counterfeit and adulterated TCM constitutes a serious threat to clinical safety and public health (Lord et al., 2001). Adulteration in the TCM market can be broadly classified into three categories (Lau et al., 2003): 1) substitution with morphologically similar species; (2) cost-driven adulteration by incorporating foreign substances; and (3) post-harvest treatments, including sulfur fumigation and artificial coloring to improve visual appeal. The high morphological similarity between adulterants and authentic specimens complicates visual authentication, particularly in the absence of standardized evaluation criteria. The incorporation of AI techniques into authenticity assessment has enhanced objectivity, analytical throughput, and reproducibility in TCM authentication practices.

*Ziziphus jujuba* Mill. var. *spinosa* (Bunge) Hu ex H. F. Chou (ZZS), which features a chemically diverse profile with multiple metabolites reported to have sedative or hypnotic potential, has experienced growing demand and price inflation, which has economically incentivized its adulteration using *Ziziphus mauritiana* Lam. (ZMS) and *Hovenia acerba* Lindl. (HAS) (Ren et al., 2023; Yang et al., 2023). To address this issue, Li et al. (2023) established an authentication model integrating Fourier Transform Near-Infrared Spectroscopy (FT-NIR) with multivariate statistical analysis. By incorporating pattern recognition algorithms—namely SVM, KNN, and ANN—the model achieved a significant improvement in identification accuracy, increasing from 77.06% to 97.58%.

In response to complex adulteration forms such as synthetic dyeing and sulfur fumigation, integrated spectroscopic and algorithmic approaches have demonstrated superior precision and analytical robustness. In a representative study, Zhang L. et al. (2023) addressed the challenge of counterfeit *Crocus sativus* L., which had been fraudulently dyed to mimic the distinctive red hue of authentic material. Using surface-enhanced Raman spectroscopy (SERS) in combination with ML algorithms—including Partial Least Squares Regression (PLSR), SVM, and Sparrow Search Algorithm based BP Neural Network (SSA-BP)—the authors developed a quantitative detection model capable of identifying both the type and concentration of added dyes. Husaini et al. (2022) combined CNNs with a Foldscope—a portable optical microscope—to create a mobile platform for saffron authentication. This lightweight yet robust system delivered markedly higher classification accuracy than conventional ML approaches such as SF and SVM. Similarly, Zhang et al. (2016) utilized hyperspectral imaging (HSI) in conjunction with chemometric analysis to identify *C. sativus* L. subjected to illicit sulfur fumigation. PCA was applied to extract key spectral features, while partial least squares discriminant analysis (PLS-DA) enabled precise classification, achieving a sensitivity of 96.4% and a specificity of 98.3%. Together, these studies provide compelling technical support for the establishment of standardized detection protocols in TCM processing and quality assurance.

To address complex adulteration scenarios, Li H. et al. (2024) developed a multimodal detection framework that integrates Artificial Intelligence Sensory (AIS) technology with Multisource Information Fusion (MIF), enabling simultaneous analysis across multiple sensory modalities. By combining data from ET, E-nose, and computer vision system, the authors constructed a comprehensive quality assessment model for *Panax notoginseng* (Burk.) F.H.Chen powder (PNP). Under controlled laboratory conditions, this model achieved a classification accuracy of 100%, underscoring its potential as a precise and reliable authentication tool for TCM quality control.

#### 3.1.4 Classification of TCM varieties

Accurate species identification of Chinese medicinal materials is critical for safeguarding clinical efficacy and advancing the standardization and modernization of TCM research (Malik et al., 2022). Conventional morphological identification methods, which rely heavily on empirical observation, are inherently subjective and low-throughput, rendering them insufficient for distinguishing morphologically similar species or processed medicinal products (Li et al., 2016; Chen et al., 2021). *Fritillaria thunbergia* Miq., for instance, exhibits substantial germplasm diversity, which in turn leads to pronounced interspecific variation in its bioactive metabolites (Nile et al., 2021). To address this challenge, Kong et al. (Kabir et al., 2022b) developed a classification model that integrates HSI with CNN to discriminate among 12 *Fritillaria* species, achieving higher cross-validation accuracy than conventional approaches.

In parallel, infrared spectroscopy coupled with chemometric analysis has emerged as a robust, non-destructive analytical approach for quality evaluation in spectroscopic studies (Cheng, 2003; Cheng et al., 2004). Li et al. (2022d) utilized attenuated total reflectance–Fourier transform infrared spectroscopy (ATR-FTIR) in conjunction with PLS-DA to distinguish 10 species of *Ophiocordyceps sinensis* (Berk.) G.H. Sung, J.M. Sung, Hywel-Jones and Spatafora with high accuracy. This method has also demonstrated effectiveness in the classification of other medicinal TCM, including *Houttuynia cordata* Thunb., *Mentha haplocalyx* Briq., *Andrographis paniculate* (Burm. f.) Nees, and *D. officinale* Kimura et Migo (Wang et al., 2018; Song et al., 2025). DL techniques have been increasingly applied to image-based recognition tasks in TCM, particularly for the analysis of macrostructural characteristics of medicinal materials. In response to the frequent confusion in species identification of *Zanthoxylum bungeanum* Maxim., Tan et al. (2024) established a CNN–based recognition model capable of differentiating multiple species with a classification accuracy of 99.35%.

#### 3.1.5 Identification of origin

Geo-authentic medicinal materials refer to TCM cultivated in defined ecological zones, where unique environmental conditions contribute to consistent quality and validated therapeutic efficacy (Zhao et al., 2012). Clinical evidence indicates that variations in geographical origin and seasonal factors markedly influence the secondary metabolite composition of a given species, potentially altering its pharmacodynamic properties (Yang et al., 2018; Miao et al., 2023). Consequently, the establishment of scientifically validated origin traceability technologies is critical for ensuring batch-to-batch consistency in the quality of Chinese medicinal materials.

HSI enables the simultaneous acquisition of spatial and spectral data, allowing non-destructive analysis of both morphological traits and chemical signatures. It has become a widely adopted tool for rapid origin identification of TCM. Recent advances have shown that combining HSI with DL algorithms can substantially enhance the accuracy of geographic origin classification for medicinal TCM. For instance, HSI integrated with CNN has been used to differentiate *Chrysanthemum morifolium* Ramat. samples from 14 distinct production regions, yielding markedly higher classification performance than conventional methods (Cai et al., 2023). Similarly, NIR-HSI coupled with ML classifiers—such as SVM and RF—has demonstrated strong robustness in distinguishing Lilium species from different origins, with notable generalizability across geographically diverse datasets (Zhao et al., 2024). In another study, He et al. (2024) combined HSI, nuclear magnetic resonance (NMR), and ResNet-34 DL framework to classify the origin of *Lilium brownii* F. E. Brown var. *viridulum* Baker, with the optimized model achieving an accuracy of 95.63%. Likewise, Cai (2023) extracted spectral features from both the visible–near-infrared (VNIR) and NIR bands to develop an attention-enhanced CNN model for the origin authentication of *Paeonia lactiflora* Pall., which significantly outperformed traditional classifiers—including KNN, RF, and SVM—in both accuracy and robustness.

Beyond HSI-based methods, infrared spectroscopy coupled with ML algorithms has also been successfully applied to origin identification of diverse medicinal TCM, such as *Bos taurus domesticus* Gmelin, demonstrating high classification performance and reliable inter-regional generalization (Wei et al., 2024). Although still in an early stage of development, E-nose technology has shown promising potential for geographic origin determination based on volatile metabolite profiles. For example, E-nose data combined with ML algorithms have been used to differentiate the origin of *Tetrastigma hemsleyanum* Diels et Gilg.,illustrating the feasibility of volatile-based traceability for TCM species (Wu et al., 2022).

#### 3.1.6 Grade evaluation

Grading evaluation of TCM is essential for ensuring clinical efficacy and maintaining batch-to-batch consistency, and is traditionally performed by quantifying selected active metabolites (Zhang and Wang, 2023; Lin et al., 2024). In recent years, the integration of histological analysis, metabolomics, and ML has introduced a novel, data-driven paradigm for TCM quality grading. For instance, the Chinese Pharmacopoeia designates astragaloside IV and calycosin-7-O-glucoside as quality control markers for *Astragalus membranaceus* (Fisch.) Bge. (AR). While sufficient for meeting Pharmacopoeia standards, these markers alone are inadequate for distinguishing between different quality grades. Wu et al. (2024) integrated metabolomic profiling with 7 ML algorithms to identify discriminatory metabolites and construct a robust classification framework for quality grading. Among the identified metabolites, amino acids—such as alanine and phenylalanine—emerged as key markers for grade differentiation, while long-chain fatty acids, including behenic acid and lignoceric acid, were critical for distinguishing wild from cultivated sources. This integrative analytical strategy has also been applied to rare and economically valuable medicinal materials. Li et al. (2022c) developed a classification model for *B. taurus domesticus* Gmelin by combining transcriptomic and metabolomic datasets, enabling reliable discrimination between natural *B. taurus domesticus* Gmelin and synthetic substitutes.

For rapid quality assessment, attenuated total reflectance–Fourier transform infrared (ATR-FTIR) spectroscopy combined with ML was applied to classify Gastrodia elata powder into four distinct quality levels. Compared with HPLC-based protocols, this approach offered higher efficiency, reduced labor requirements, and allowed for non-destructive testing (Zhan et al., 2022).

Furthermore, for the detection of internal defects, the integration of X-ray imaging with a YOLOv5 deep learning architecture substantially improved sensitivity and accuracy in identifying internal cavities and pest-induced damage in Panax ginseng, significantly outperforming conventional manual inspection methods (Boniecki et al., 2014; Xue et al., 2023).

#### 3.1.7 Identification of storage age

The traditional adage, “For a 7-year illness, seek three-year-old moxa wool (MW),” reflects the critical role of storage duration in shaping the therapeutic efficacy of TCM (Xue et al., 2020). Extended storage can markedly alter the VOCs of MW, with potential consequences for its pharmacological activity. Therefore, precise determination of the storage period for TCM materials is essential to ensure product quality, preserve chemical stability, and promote standardization in both clinical practice and industrial production.

Recent studies have shown that combining HSI with ML provides a powerful non-destructive approach for determining the storage age of TCM. For instance, Hu et al. (Hu et al., 2024) developed a rapid classification model for MW with varying storage durations by integrating HSI data with ML algorithms. The optimized model achieved classification accuracies of 99.78% in the VNIR range and 99.47% in the short-wave infrared (SWIR) range, offering a practical, non-invasive solution for rapid quality assessment based on storage-dependent spectral signatures. Similarly, for *C. reticulata* Blanco (CRP), HSI data acquired in the 874–1734 nm range were combined with an extreme learning machine (ELM) classifier to differentiate samples stored for 1, 5, 10, and 15 years. The resulting model achieved an accuracy exceeding 85%, confirming the feasibility of the HSI–ELM approach for storage-age classification of CRP (Li et al., 2024c). In parallel, E-nose systems have demonstrated considerable potential for distinguishing *Lonicera japonica* Thunb. samples by capturing storage-dependent odor fingerprints, thereby offering a complementary, sensory-based modality for non-destructive quality evaluation (Xiong et al., 2014).

#### 3.1.8 Analysis of TCM metabolites

Advances in AI provide sophisticated tools for identifying chemical metabolites of TCM, thereby improving the precision and efficiency of metabolite analysis and deepening mechanistic understanding. In particular, AI-assisted interpretation of chromatographic fingerprints and spectroscopic data (e.g., mass spectrometry and nuclear magnetic resonance) enables rapid, accurate identification of metabolites within TCM and complex formulations. For example, Guo et al. (2021) integrated DL with UHPLC-Q-TOF/MS to enhance the chemical profiling of Qianghuoshengshi decoction; the model achieved TCM-specific classification of coumarins and chromones, guided identification via characteristic ions and neutral-loss patterns, and produced fingerprints that supported sensitive, multi-target quantification by UHPLC-sMRM. Building on this theme, Zuo et al. (2021) orthogonally optimized LC–QTOF/MS conditions to maximize metabolite coverage in *Gelsemium elegans* (Gardn.&Champ.) Benth., and then implemented an AI-assisted data-mining pipeline—database-guided annotation combined with diagnostic-ion and neutral-loss filters—to automate high-intensity chemical profiling. Collectively, these studies illustrate how rule-based AI embedded in MS data mining accelerates metabolite discovery while enabling validated quantitation in complex TCM matrices.

Beyond organic-metabolite profiling, AI also facilitates the identification, classification, and prediction of elemental fingerprints in TCM (Zhang and Wang, 2023; Ding et al., 2024a). For instance, Zhao Y. et al. (2018) compared 13 trace elements and caffeoylquinic-acid–based actives in *Lonicera confusa* (Sweet) DC. and *L. japonica* Thunb. by using PCA and DA and achieved clear interspecies classification. Wei et al. (2024) combined NIR with SVM regression to non-destructively quantify seven active metabolites and elements in *Cornus officinalis* Sieb. et Zucc., while ICP-AES multi-element profiling plus correlation analysis demonstrated stronger associations between in-sample inorganic elements and active metabolites than with rhizosphere soil elements and revealed notable K/Ca enrichment patterns.

#### 3.1.9 Process optimization

Variability in TCM processing remains common due to unstandardized operations, imprecise temperature control, and metabolite loss, leading to inconsistent quality (Kouadio et al., 2024; Zhang J. et al., 2024). Batch-to-batch uniformity and efficiency are also difficult to maintain under manual workflows (Ni et al., 2020). AI coupled with big-data analytics and model training has been applied to processing and quality control. Procedures can be optimized, quality monitored, metabolite consistency supported, and production automated, thereby improving overall quality and efficacy (Zhang et al., 2020).

At raw-material procurement, big-data analyses have been used to evaluate origin, season, and climate effects on TCM quality (Xu et al., 2023). Predictive models trained on historical sources and standards can rank high-quality suppliers and flag noncompliant lots, informing purchasing decisions (Ameer et al., 2020).

During concoction, AI-assisted optimization of technique selection and parameters has been reported to enhance efficacy and reduce adverse reactions (Chen H. et al., 2024). ML has been used to analyze the effects of frying, roasting, and calcining on active metabolites (Kang et al., 2016). Processing conditions—temperature, humidity, and time—can be automatically optimized. For example, E-nose and NIR combined with AI have been used to detect internal and surface changes during *Curcuma longa* L. processing. Liu Q. et al. (2025) employed HSI for nondestructive monitoring of jujube quality during hot-air drying and built deep-learning models that accurately predict dried-product attributes. Historical and experimental data have also been used to predict and adjust parameters in real time, reducing thermal degradation of active metabolites at high temperatures (Liu et al., 2023).

### 3.2 AI-driven mechanistic evaluation using bioinformatics

The therapeutic efficacy of TCM often derives from synergistic interactions that cannot be fully explained by quantifying a limited subset of chemical metabolites. The integration of bioinformatics with AI has notably facilitated the development of a mechanism-oriented quality control framework, offering a forward-looking pathway toward the scientific modernization of TCM.

**FIGURE 6:**
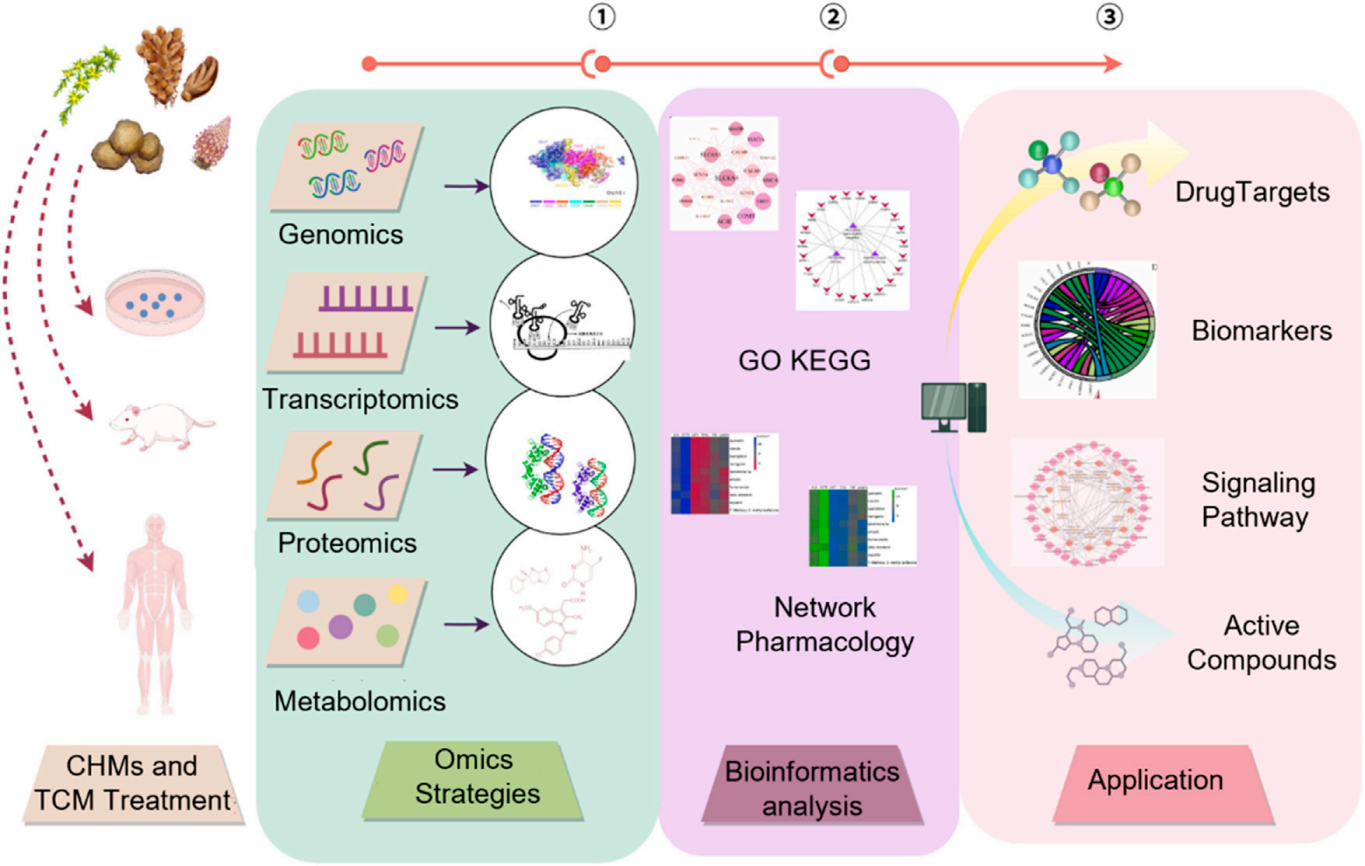
Applications of AI and bioinformatics in TCM.

This emerging paradigm emphasizes the creation of a causality-driven system that seamlessly links the identification of Q-markers, the elucidation of therapeutic targets and detailed mechanistic analysis. By integrating transcriptomic, metabolomic, and network-level datasets with advanced intelligent algorithms, the framework enables automated prediction of pharmacological mechanisms, target profiles, and potential toxicity risks (Figure 6). Table 2 summarizes typical cases of the application of AI combined with various technologies in the mechanism evaluation of TCM.

**TABLE 2:** Representative studies on the application of AI in the mechanistic evaluation of TCM.

Main research contents	Research objective	Substance types	Algorithms	References
Identification of Q-markers	Identify Q-markers from *Hypericum perforatum L*. for quality assessment	UPLC-Q-TOF-MS/MS; OPLS-DA, network-based analysis	SVM; KNN; RF	Zhang et al. (2024c)
Identification of Q-markers	Predict novel bioactive metabolites from TCM	Network pharmacology; molecular docking	DeepDGC	Fu et al. (2023)
Identification of Q-markers	To discover candidate for inhibiting hepatocellular carcinoma from *Phytolacca acinose Roxb*	Transcriptome sequencing; molecular docking, molecular dynamics; biological network analysis	DL; SNF	Liu et al. (2024b)
Elucidation of mechanisms	To investigate the anti-osteoporotic mechanism of curcumin	Network pharmacology; multi-omics integration	data mining	Chen et al. (2024b)
Elucidation of mechanisms	To elucidate the mechanism of *Scutellaria baicalensis* Georgi in treating NSCLC	Network pharmacology; bioinformatics; radiomics	ML	Su et al. (2025)
Elucidation of mechanisms	Develop a prediction model for HILI and explore toxicity mechanisms	HILI database	ML-based predictive modeling	Wu et al. (2019)
Elucidation of mechanisms	Analyze the pharmacological mechanism of Xiaoxuming decoction	Pharmacological data	NB network	Yang et al. (2019)
Exogenous toxicity mechanism prediction	Elucidate the mechanism of hepatotoxicity of TW	UPLC-Q-TOF-MS; network toxicology; Western blot	Mechanistic network construction	Zhou et al. (2025)
Exogenous toxicity mechanism prediction	To investigate the hepatoprotective effect of SC against TW-induced toxicity	Network pharmacology; molecular docking; qRT-PCR; Western blot	Mechanistic modeling	Ji et al. (2025)
Exogenous toxicity mechanism prediction	To predict hepatotoxic metabolites in Polygonum multiflorum Thunb	TCM-induced liver injury dataset	Ensemble ML classifier	He et al. (2019)
Exogenous toxicity mechanism prediction	To screen TCM-related metabolites for nephrotoxicity	QSAR model based on 609 metabolites	ANN; SVM	Sun et al. (2019)
Exogenous toxicity mechanism prediction	To improve high-throughput detection of pesticide residues under structural ambiguity	UV-Vis spectroscopy	Transformer deep learning architecture	Zhang et al. (2025a)
Exogenous toxicity mechanism prediction	To achieve ultrasensitive visual detection of pesticide residues	SERS with silver nanoparticles	1D-CNN; other CNN architectures	Wang et al. (2025)

#### 3.2.1 Efficacy-oriented evaluation based on pharmacological mechanisms

The modernization of TCM efficacy evaluation calls for a decisive transition from the conventional model—rooted in metabolite quantification and empirical judgment—to a precision-oriented framework anchored in pharmacologically relevant mechanisms. This shift depends on the identification of biomarkers that are closely linked to therapeutic efficacy and on the elucidation of pharmacodynamic mechanisms.

##### 3.2.1.1 Identification of Q-markers

Reliable biomarkers that accurately capture the pharmacological effects of TCM are essential for precision quality control. Unlike conventional indicators—typically derived from quantitative chemical assays or empirical selection—modern evaluation frameworks place greater emphasis on Q-markers that are directly linked to therapeutic efficacy and possess clearly defined pharmacological functions (Zhou et al., 2023).

For example, *Hypericum perforatum* L., which exhibits pronounced chemical variation across its medicinal parts, has been studied using ultra-performance liquid chromatography coupled with quadrupole time-of-flight tandem mass spectrometry (UPLC-Q-TOF-MS/MS), orthogonal projections to latent structures discriminant analysis (OPLS-DA), and network-based analytical approaches. Through these methods, bioactive differential metabolites were identified as candidate Q-markers and subsequently validated using ML models—including SVM, KNN, and RF—confirming their utility in quality assessment (Zhang Z. et al., 2024). Fu et al. (2023) developed DeepDGC, a DL–based framework that integrates network pharmacology with molecular docking to predict novel bioactive metabolites. Among the predicted candidates, glabrone and vestitol showed binding affinity for SARS-CoV-2–associated proteins and modulated inflammation-related targets such as PTEN and MAP3K8, highlighting their potential as Q-markers.

Moreover, integrating DL with similarity network fusion (SNF) has shown substantial promise in elucidating the mechanisms underlying complex diseases. For instance, in assessing the hepatocellular carcinoma–inhibitory potential of *Phytolacca acinose* Roxb., Liu et al. (Liu J. et al., 2024) combined biological network analysis, transcriptome sequencing, molecular docking, and molecular dynamics simulations to identify xanthomicrol as a promising therapeutic candidate. Subsequent *in vivo* experiments not only validated its antitumor efficacy but also clarified its molecular mechanism of action. Collectively, this integrative research framework exemplifies a representative paradigm for Q-markers studies in TCM, seamlessly bridging predictive modeling, mechanistic elucidation, and experimental validation.

##### 3.2.1.2 Elucidation of pharmacodynamic mechanisms

TCM is characterized by its inherently multi-target and multi-pathway therapeutic strategies. While such characteristic have yielded substantial clinical benefits, they also present considerable challenges in elucidating the underlying mechanisms (Zhai et al., 2019). The long-standing issues of “unclear mechanisms” and “undefined targets” remain central points of critique toward TCM (Guo et al., 2019). In this context, integrating AI with multi-omics technologies and network pharmacology provides a robust and systematic approach to dissect the complex pharmacodynamic networks underlying TCM interventions. This, in turn, enables a more precise and evidence-based interpretation of their therapeutic mechanisms. A representative example is the mechanistic investigation of curcumin in the treatment of osteoporosis (OP). Chen S. et al. (2024) employed bioinformatics and data mining techniques to examine the involvement of ferroptosis in OP, with the aim of identifying key regulatory factors. Their analysis revealed MAPK3, TGFB1, CYBB, EGFR, and PTGS2 as hub genes closely linked to ferroptosis, offering novel insights into the molecular basis of curcumin’s anti-osteoporotic effects. Subsequent analysis revealed that curcumin modulates iron homeostasis via EGFR and PTGS2, supporting its potential therapeutic role in OP management.

In cancer therapy, Su et al. (2025) conducted a comprehensive analysis of the therapeutic potential of *Scutellaria baicalensis* Georgi in treating non-small cell lung cancer (NSCLC) through the integration of network pharmacology, bioinformatics, ML, and radiomics. They constructed an “active metabolite–target–disease” (ATD) network and, through computational analysis, identified five core targets—FABP4, XDH, GPBAR1, CA4, and CDH1—as pivotal nodes within this network. This study elucidated the multi-target pharmacological mechanisms of S. baicalensis and offered theoretical insights into personalized therapeutic strategies through a data-driven analytical framework.

In TCM toxicology, Wu et al. (2019) created a prediction model using the TCM-induced liver injury (HILI) database to support large-scale screening and explore toxicity mechanisms. Yang et al. (2019) used Bayesian network to analyze the pharmacological mechanism of Xiaoxuming decoction. The model demonstrated robust performance in characterizing the pharmacological profiles of multi-metabolite formulations, highlighting the utility of probabilistic inference methods in elucidating complex TCM mechanisms.

With advances in AI and bioinformatics, TCM research is undergoing a transformation from empirical knowledge to data-driven analysis. This shift includes analysis of both single TCMs and complex prescriptions, and expands from single-target prediction to network-level mechanism reconstruction.

#### 3.2.2 Safety assessment driven by toxicological mechanisms

The safety of TCM is fundamental to its clinical application and global acceptance. Nevertheless, TCM safety evaluation still faces several critical challenges, such as the complex and variable nature of toxic metabolites, the absence of clearly defined toxic thresholds, the limited understanding of detoxification mechanisms, and the lack of precise and standardized detection technologies. Conventional safety assessments primarily depend on animal experimentation and empirical judgment, which fail to elucidate the causal links between endogenous and exogenous toxicants in TCM and their corresponding target organs or toxicological pathways. To meet the urgent needs and technical challenges in TCM safety evaluation, it is crucial to enhance fundamental research both internally and externally, with particular emphasis on in-depth exploration and comprehensive understanding of the biological mechanisms underlying toxic effects. In this context, the integration of bioinformatics, multi-omics technologies, and AI has facilitated the establishment of a multi-dimensional safety evaluation framework encompassing mechanistic toxicology, predictive modeling, and risk screening.

##### 3.2.2.1 Mechanistic prediction of endogenous toxic metabolites

Numerous natural bioactive metabolites in TCM possess intrinsic toxicity, among which hepatotoxicity and nephrotoxicity are the most frequently observed, raising considerable safety concerns. The integration of modern multi-omics approaches with network toxicology offers a robust strategy for identifying key toxic metabolites, their molecular targets, and associated signaling pathways. Zhou et al. (2025) used untargeted metabolomics based on ultra-high performance liquid chromatography with quadrupole time-of-flight mass spectrometry (UPLC-Q-TOF-MS) combined with network toxicology and Western blotting, to reveal that the hepatotoxicity of *Tripterygium wilfordii* Hook. f. (TW) involves multiple signaling pathways and abnormal protein expression. They also constructed a mechanistic network linking metabolites to their target proteins. In clinical practice, *Spatholobus suberectus* Dunn (SC) has been reported to alleviate the adverse effects induced by TW. Ji et al. (2025) explored the hepatoprotective mechanisms of SC through network pharmacology and molecular docking, followed by experimental validation using quantitative real-time PCR (qRT-PCR) and Western blot analysis. The results demonstrated that SC mitigates TW-induced hepatotoxicity by inhibiting the HIF-1α/VEGFA signaling axis and lowering triptolide levels, while preserving its anti-inflammatory efficacy (He et al., 2020; Sun S. et al., 2022).

AI has emerged as a crucial tool in advancing TCM quality and safety evaluation, particularly in the prediction of hepatotoxicity and nephrotoxicity. He et al. (2019) developed a large-scale dataset on TCM-induced liver injury and applied multiple ML algorithms to construct an ensemble classifier, which identified 25 potentially hepatotoxic metabolites in *Polygonum multiflorum* Thunb. Sun et al. (2019) established a quantitative structure–activity relationship (QSAR) model based on 609 metabolites, including natural products, modern drugs, and hybrid datasets. ANN and SVM algorithms were applied for nephrotoxicity prediction, and validation demonstrated the highest accuracy in the natural product subset, with ANN and SVM achieving 96.7% and 93.3%, respectively. These modeling approaches provide practical tools and valuable references for screening TCM-related metabolites for nephrotoxicity and for evaluating the toxicological profiles of natural products.

AI has emerged as a crucial tool in advancing TCM quality and safety evaluation, particularly in the prediction of hepatotoxicity and nephrotoxicity. He et al. [106] developed a large-scale dataset on TCM-induced liver injury and applied multiple ML algorithms to construct an ensemble classifier, which identified 25 potentially hepatotoxic metabolites in Polygonum multiflorum Thunb. Sun et al. [107] established a quantitative structure–activity relationship (QSAR) model based on 609 metabolites, including natural products, modern drugs, and hybrid datasets. ANN and SVM algorithms were applied for nephrotoxicity prediction, and validation demonstrated the highest accuracy in the natural product subset, with ANN and SVM achieving 96.7% and 93.3%, respectively. These modeling approaches provide practical tools and valuable references for screening TCM-related metabolites for nephrotoxicity and for evaluating the toxicological profiles of natural products.

##### 3.2.2.2 Mechanistic studies of exogenous toxic metabolites

Exogenous hazardous substances—such as heavy metals and pesticide residues—are commonly present during the cultivation, harvesting, processing, and storage of medicinal TCMs, posing substantial risks to the safety and quality control of TCM. These substances can interact complexly with the bioactive or toxic metabolites of TCM, potentially interfering with therapeutic efficacy or exacerbating toxicity. Therefore, elucidating the toxicological mechanisms of exogenous substances in the context of TCM is essential for ensuring medicinal safety and advancing TCM modernization.

Heavy metal contaminants—such as As, Cd, Pb, and Hg—commonly detected in medicinal materials primarily arise from plant bioaccumulation, cross-contamination during processing, and the application of heavy metal–containing therapeutic agents or metalloids (Nagarajan et al., 2014b; 2014a). Integrating ionomics, metabolomics, and transcriptomics, researchers have shown that Cd induces neurotoxicity and multi-organ dysfunction through disruption of metabolic pathways and alteration of gene expression. Meanwhile, Se has shown significant protective and detoxifying effects, alleviating Cd-induced toxicity (Zhang X. et al., 2023). Xie et al. (2023) further employed spatially resolved metallomics to systematically map the Se distribution in seeds of the hyperaccumulator plant *Cardamine violifolia* O.E. Schulz, revealing selenium-associated tolerance mechanisms. This study offers theoretical insights into metalloid accumulation and detoxification mechanisms in medicinal TCMs.

For pesticide residue detection, conventional methods—such as spectrophotometry, GC, thin layer chromatography (TLC), and HPLC—generally provide sufficient sensitivity and specificity. However, their application to metabolites with undefined or poorly characterized structures remains limited, primarily due to restricted analytical throughput and heavy reliance on predefined molecular targets (Xiong et al., 2018; Pan et al., 2022; Wang et al., 2023). To address these challenges, AI-enhanced spectroscopic strategies have emerged as promising alternatives. For example, Zhang H. et al. (2025) integrated UV-Vis spectroscopy with a Transformer deep learning architecture, where the self-attention mechanism captured complex spectral dependencies and resolved overlap, offering a feasible approach for high-throughput pesticide residue detection in cases of structural ambiguity. Moreover, to overcome these limitations, researchers have established a multifunctional SERS–based detection system for the simultaneous visualization and quantification of pesticide residues. The system employs silver nanoparticles as the SERS substrate and combines vertex metabolite analysis with the Euclidean distance algorithm to achieve ultrasensitive visual detection of pesticide residues (Wang et al., 2025). Building on this, SERS has the potential to be integrated with AI algorithms—such as one-dimensional convolutional neural networks (1D-CNN) and other CNN architectures—to enable precise identification, classification, and quantification of multiple pesticide residues (Li et al., 2021; Zhu et al., 2021; Wang et al., 2024). The emergence of these “SERS + AI” hybrid models has enhanced the efficiency and sensitivity of pesticide residue detection in medicinal TCMs, while providing advanced tools and theoretical foundations for developing intelligent quality control systems in accordance with modern TCM quality standards.

## 4 Advantages and challenges

### 4.1 Advantages

**FIGURE 7:**
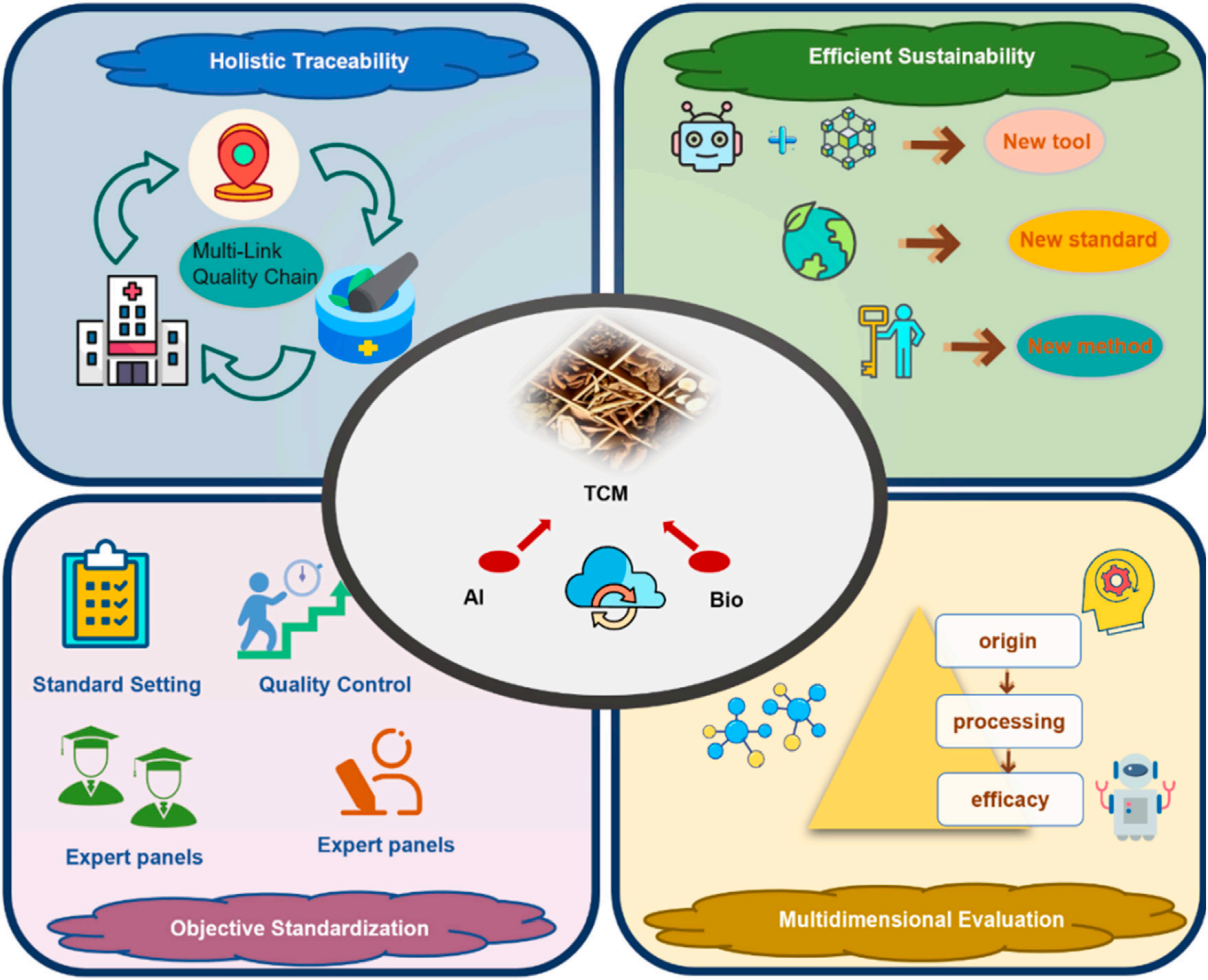
Advantages of the integrated strategy of AI and bioinformatics.

The integration of AI and bioinformatics into TCM quality control provides systematic, data-driven solutions to overcome the limitations of conventional methods. As shown in Figure 7, these advantages are mainly reflected in the following aspects:

1. Enhancing holistic understanding of TCM and supporting multi-link quality traceability

AI can be applied across the full TCM lifecycle, from cultivation to storage, and concurrently support mechanistic analyses to safeguard clinical safety and efficacy. By modeling multidimensional data—such as environmental factors, growth traits, and phytochemical profiles—these tools help identify pharmacologically active metabolites and intrinsic Q-markers. This supports the development of evaluation systems with features specific to TCM (Yang et al., 2022; Kousar et al., 2023). Crucially, along the time axis, AI consolidates what conventional non-AI workflows treat as sequential, manual steps—literature triage, feature curation, and repeated chromatographic re-runs—into a front-loaded model training phase followed by millisecond-to-second per-sample inference, thereby shortening batch turnaround and reducing reviewer time in routine quality control.

2. Enhancing research efficiency, reducing costs, and supporting environmentally sustainable development

AI algorithms can rapidly identify pharmacologically active and potentially toxic metabolites from large-scale datasets, thereby streamlining and accelerating fundamental TCM research. In the investigation of rare medicinal TCMs or complex formulations, AI reduces iterative experimental cycles, shortens research timelines, and minimizes reliance on animal testing, in accordance with ethical and sustainability principles (Liu T. et al., 2025). Relative to conventional trial-and-error or rule-based screening, once trained, AI yields lower per-sample operating cost and markedly faster inference throughput, shifting costs from repeated assays to one-time model development.

3. Enhancing objectivity in quality control and promoting standardization

Traditional identification hinges on inspector expertise, introducing subjectivity and variability. Incorporating ML enables advanced analysis of high-throughput imaging and spectroscopic profiles, improving the objectivity and reproducibility of assessments (Ding et al., 2024b). Under external validation, AI models generally show higher accuracy and better cross-batch robustness than non-AI chemometric baselines, providing firmer ground for standardization and inter-lab transfer. In addition, aligning mechanisms with quality models establishes a robust scientific basis for standardized quality control.

4. Supporting multidimensional quality evaluation aligned with the holistic nature of TCM formulas

AI and bioinformatics enable multidimensional evaluation of TCM quality—spanning metabolites, targets, and pathways—addressing the limitations of single-parameter models. This better reflects TCM’s intrinsic characteristics (multiple metabolites, diverse targets, interconnected pathways). In practice, AI supports scalable fusion of origin, growth duration, and mechanistic readouts, yielding more comprehensive and clinically applicable quality attributes than conventional one-factor approaches (Li et al., 2022e; 2024b).

### 4.2 Challenges

Despite their promising applications in TCM quality control, AI and bioinformatics face several practical challenges, summarized as follows:

1. Data standardization and model reliability require further improvement

The effectiveness of AI and bioinformatics models relies heavily on high-quality, standardized, and representative datasets. However, in the context of TCM, heterogeneous data types, outdated omics databases, and inconsistent metadata annotations significantly reduce model performance, reproducibility, and cross-context generalizability. In addition, AI-based predictions often lack mechanistic interpretability, highlighting the need for biological validation to enhance reliability and reduce the risks posed by opaque “black-box” models that may mislead scientific conclusions (Messeri and Crockett, 2024).

2. Insufficient privacy protection mechanisms

TCM-related clinical and multi-omics datasets often contain sensitive personal information (Jin and Qin, 2021). However, a comprehensive, TCM-specific data governance framework for privacy protection remains underdeveloped. AI model training poses significant risks of patient data leakage and unauthorized use. Moreover, data-sharing mechanisms on bioinformatics platforms require further refinement under strict regulatory and ethical standards (Khalid et al., 2023).

3. Limited adaptability of current models

The inherent complexity of TCM, characterized by multi-metabolite formulations and nonlinear multi-target interactions, poses substantial challenges to the design and optimization of intelligent predictive models. At present, dedicated algorithms and predictive frameworks capable of systematically capturing multi-metabolite synergy in TCM remain underdeveloped. This deficiency impedes the advancement of intelligent research architectures that are consistent with the holistic and integrative therapeutic principles of TCM (Jiang et al., 2025).

4. Practical barriers to AI implementation in TCM production and regulatory

In TCM quality control, AI faces practical obstacles on production and regulatory contexts. The lack of demonstrated method equivalence and commutability under real raw-material variability (origin, season, processing) prevents AI outputs from replacing pharmacopeial release tests. Workflow integration remains fragile, with predictions not consistently mapped to SOP decision points or written to LIMS/MES and electronic batch records, limiting timely batch disposition. Transferability and model drift across instruments and sites necessitate frequent recalibration and external-batch re-validation, raising operational burden. Regulatory acceptance is further constrained by incomplete evidence packages—predefined statistical criteria, multicenter comparability, and audit-ready data lineage—together with insufficiently actionable explainability that links model attributions to pharmacopeial peaks or Q-markers. Finally, change control and potential re-approval for model updates, coupled with uncertain return on investment and limited analyst training, slow sustained adoption.

5. Key advantages and obstacles for explainable AI in TCM clinical and production

Explainable artificial intelligence can improve trust in TCM clinical and production settings. It links model rationales to pharmacopeial thresholds and SOP decision points. It also communicates calibrated reasoning and uncertainty and records traceable outputs in LIMS, MES, and electronic batch records. However, implementation remains constrained. Heterogeneous fingerprints and spectral collinearity can distort *post hoc* explanations such as partial dependence and Shapley attribution. Many explanations lack monotonic or shape constraints that match process windows. Communication of uncertainty and distribution shift is often inadequate. Standardized external validation remains limited, including fidelity, stability, calibration, and violation rates. Links to laboratory and manufacturing records are not consistently traceable. Human-in-the-loop triggers and change control with revalidation are immature. These issues temper adoption despite clear potential benefits.

## 5 Conclusion and prospect

In recent years, various AI-driven strategies have shown promising results in addressing core challenges in TCM quality control. As summarized in the preceding sections, representative ML and DL systems have been evaluated across three key dimensions: First, statistical validation employed cross-validation protocols—often nested—and bootstrap confidence intervals, with head-to-head comparisons against conventional chemometric baselines to quantify incremental benefit. Second, external and transfer validation was performed using geographically and temporally independent datasets (e.g., cross-region and cross-batch acquisitions, cross-instrument splits), demonstrating generalization under realistic shifts in fingerprint heterogeneity and spectral collinearity; representative tasks reported high discriminative performance in external settings. Third, clinical and regulatory alignment was addressed by anchoring model thresholds to pharmacopeial quality indices and predefined Q-markers ranges, and—where available—linking predictions to operational or clinical endpoints such as batch-release pass rate, rework rate, and turnaround time.

Collectively, these layers provide adoption-relevant evidence—rigorous statistics, external testing, and outcome linkage—that bridge algorithmic feasibility and real-world implementation, and they frame the strategic directions that follow.

Nonetheless, TCM’s unique holistic framework—centered on multi-TCM prescriptions, syndrome differentiation, and synergistic pharmacology—continues to pose system-level complexity that conventional methods cannot fully capture. Future efforts should therefore focus on the following strategic directions:

1. Building multidimensional databases to enable AI–domain knowledge co-modeling

Priority should be given to constructing a standardized, multidimensional TCM database that encompasses chemical composition profiles, target networks, toxicological pathways, multi-omics data, and clinical efficacy information. By incorporating ontologies, knowledge graphs, and domain-specific knowledge to enhance learning, AI models can be endowed with semantic understanding and reasoning capabilities in the TCM domain. This promotes the deep integration of theory and AI and facilitates the reconstruction of a closed-loop “data–knowledge–mechanism” system in TCM research.

2. Developing explainable AI and causal inference approaches to improve model transparency and trust

AI models with attention mechanisms can help dissect internal decision pathways, improving traceability and interpretability for scientific reproducibility. Incorporating causal directed acyclic graphs (causal DAGs) into bioinformatics analyses allows the identification of mechanistic targets and intervention pathways, reducing spurious predictions and enhancing biological relevance.

3. Strengthening privacy protection and ethical guidelines to build a compliant intelligent system

The application of privacy-preserving machine learning (PPML) is essential for secure and ethical TCM data sharing and model development. A multi-level, regulation-compliant ethical framework should be established to cover the entire data lifecycle—from acquisition and storage to sharing and analysis (Khalid et al., 2023).

4. Promoting visualization and low-code tools to support cross-disciplinary applications

Since most TCM researchers lack computational training, developing low-code or no-code AI platforms for biomedical applications is essential to facilitate adoption. Such tools can reduce the threshold for AI adoption and promote interdisciplinary innovation and translational research and clinical application (Sundberg and Holmström, 2023).
