# Supplementary material for: Application and research progress on artificial intelligence in the quality of Traditional Chinese Medicine
Source: Front Pharmacol. 2025 Oct 17;16:1687681. doi: 10.3389/fphar.2025.1687681 (PMC12575287; doi:10.3389/fphar.2025.1687681)
Supplement: Supplementary file 1 [file DataSheet1.pdf]

Supplementary Material

1 Supplementary Table 1

Table S1. List of botanical drugs referenced in this review

Botanical drug	Family	Standardized Pharmaceutical Latin	Processing method	Reference
Andrographis paniculata (Burm. f.) Nees	Acanthaceae	Andrographis Herba	The powdered botanical drugs, obtained by grinding and sieving with a minimum 80% sieve rate, were stored in desiccators or refrigerators.	(Song et al., 2025)
Astragalus membranaceus (Fisch.) Bge.	Fabaceae	Radix Astragali	Approximately 2.0 g of powdered AR slices were refluxed with methanol, filtered, mixed with internal standards, and centrifuged to prepare the sample for analysis.	(Wu et al., 2024)
Bos taurus domesticus Gmelin	Bovidae	Calculus Bovis	The samples were ultrasonically extracted twice with a 1% formic acid-methanol solvent, and the combined supernatants were diluted and centrifuged to obtain the final sample solution.	(Wei et al., 2024)
Boswellia carterii Birdw.	Burseraceae	Olibanum (*gummi-resina e trunco*)	The samples were quickly crushed using a low-temperature mortar, and after crushing, they were quickly sieved through a 50 mesh. The fine frankincense powder was stored in a −80 °C refrigerator.	(Chen et al., 2024)

Cardamine violifolia O.E. Schulz	Brassicaceae	Herba Cardaminis Violifoliae	The samples (20 mg) were predigested with 5 mL of HNO3 and 0.5 mL of H2O2 and left at room temperature overnight.	(Xie et al., 2023)
Chrysanthemum morifolium Ramat.	Asteraceae	Asteraceae; Chrysanthemi Flos	The chrysanthemum samples were dried to remove moisture, after which they were meticulously stored.	(Cai et al., 2023)
Citrus reticulata Blanco	Rutaceae	Pericarpium Citri Reticulatae	The study extracted volatiles of low molecular weight using steam distillation.	(Li et al., 2022a)
Citrus reticulata Blanco	Rutaceae	Pericarpium Citri Reticulatae	Not reported	(Li et al., 2024b)
Cornus officinalis Sieb. et Zucc.	Cornaceae	Corni Fructus	For HPLC analysis,the samples were extracted with 70% ethanol via ultrasonication, whereas for ICP-MS analysis, they were digested with nitric acid using microwave assistance.	(Zhao et al., 2018)
Crocus sativus L.	Iridaceae	Croci Stigma (Saffron)	Not reported	(Husaini et al., 2022)
Curcuma longa L.	Zingiberaceae	Rhizoma Curcumae Longae (Turmeric)	Not reported	(Zhao et al., 2018)
Dendrobium officinale Kimura et Migo	Orchidaceae	Dendrobii Caulis	Not reported	(Chang et al., 2024)
Dendrobium officinale Kimura et	Orchidaceae	Dendrobii Caulis	1 mg sample of powder was precisely weighed and extracted with 1.5 ml of 80% methanol using	(Wang et al.,

Migo			ultrasound after a 30-minute immersion at room temperature.	2018)
Fritillaria thunbergia Miq.	Liliaceae	Fritillariae thunbergii bulbus	Not reported	(Nile et al., 2021)
Houttuynia cordata Thunb.	Saururaceae	Houttuyniae Herba	The powdered herbs, obtained by grinding and sieving with a minimum 80% sieve rate, were stored in desiccators or refrigerators.	(Song et al., 2025)
Hovenia acerba Lindl.	Rhamnaceae	Hoveniae Semen Seu Fructus	All samples were crushed into powder by a highspeed crusher, passed through a 65-mesh sieve (250 ± 9.9um), and stored in dry, sealed, and dark environment.	(Li et al., 2023)
Hypericum perforatum L.	Hypericaceae	Herba Hyperici Perforati	0.1 g sample of HPL powder was ultrasonically extracted with 10 mL of 60% methanol, then centrifuged and filtered to obtain the test solution for analysis.	(Zhang et al., 2024)
Lilium brownii F. E. Brown var. *viridulum* Baker	Liliaceae	Fritillariae thunbergii bulbus	These berries were naturally sun-dried over a period of several days.	(He et al., 2024)
Lonicera confusa (Sweet) DC.	Caprifoliaceae	Lonicerae Flos	For HPLC analysis,the samples were extracted with 70% ethanol via ultrasonication, whereas for ICP-MS analysis, they were digested with nitric acid using microwave assistance.	(Zhao et al., 2018)
Lonicera japonica Thunb.	Caprifoliaceae	Flos Lonicerae Japonicae	All samples stored at 22±1°C away from light.	(Xiong et al., 2014)

Lonicera japonica Thunb.	Caprifoliaceae	Flos Lonicerae Japonicae	The kernel of CF was removed. The pulp was dried followed by crushing into powder and stored in the refrigerator at 4 ◦C for later use.	(Wei et al., 2024)
Mentha haplocalyx Briq.	Lamiaceae	Menthae Haplocalycis Herba	The powdered botanical drugs, obtained by grinding and sieving with a minimum 80% sieve rate, were stored in desiccators or refrigerators.	(Song et al., 2025)
Ophiocordyceps sinensis (Berk.) G.H. Sung, J.M. Sung, Hywel-Jones & Spatafora	Ophiocordycipitaceae	Cordyceps	The dried samples were pulverized into fine-grained powder and through a 60-mesh sieve and frozen stored at −20°C in a polyethylene sealing bag.	(Li et al., 2022b)
Panax notoginseng (Burk.) F.H.Chen	Araliaceae	Panax (radix et rhizoma)	All samples were powdered and then uniformly sieved using a Pharmacopoeia No. 5 sieve.	(Li et al., 2024a)
Phytolacca acinose Roxb.	Phytolaccaceae	Phytolaccae Radix	Not reported	(Liu et al., 2024)
Polygonum multiflorum Thunb.	Polygonaceae	Radix Polygoni Multiflori	Not reported	(He et al., 2019)
Scutellaria baicalensis Georgi	Lamiaceae	Scutellariae Radix	Not reported	(Su et al., 2025)
Spatholobus suberectus Dunn	Fabaceae	Caulis Spatholobi	Three different processed TW products were prepared by blending TW slices with varying proportions of concentrated SC decoction, allowing the mixture to stand, stir-frying, and then	(Ji et al., 2025)

			drying.	
Tetrastigma hemsleyanum Diels et Gilg.	Vitaceae	Radix Tetrastigmatis Hemsleyani	Not reported	(Wu et al., 2022)
Tripterygium wilfordii Hook. f.	Celastraceae	Radix Tripterygii Wilfordii	The dried and powdered TW medicinal materials were extracted with ethanol, and the resulting extract was concentrated, freeze-dried, and then formulated into suspension solutions with 0.5% CMC-Na for sampling.	(Zhou et al., 2025)
Zanthoxylum bungeanum Maxim.	Rutaceae	Zanthoxyli Pericarpium	Not reported	(Tan et al., n.d.)
Ziziphus jujuba Mill. var. *spinosa* (Bunge) Hu ex H. F. Chou	Rhamnaceae	Ziziphi Spinosae Semen	All samples were crushed into powder by a highspeed crusher, passed through a 65-mesh sieve (250 ± 9.9um), and stored in dry, sealed, and dark environment.	(Li et al., 2023)
Ziziphus mauritiana Lam.	Rhamnaceae	Ziziphi Mauritianae Semen	All samples were crushed into powder by a highspeed crusher, passed through a 65-mesh sieve (250 ± 9.9um), and stored in dry, sealed, and dark environment.	(Li et al., 2023)

2 Supplementary Table 2

Table S2. Formula composition, drug ratios, and extraction parameters

Name of TCM	The botanical drugs of TCM	Processing method	Reference
Qianghuoshengshi decoction (QHSS)	QHSS is composed of seven botanical drugs including Angelicae Pubescentis Radix (APR), Notopterygii Rhizoma et Radix (NRR), Ligustici Rhizoma et Radix (LRR), Saposhnikoviae Radix (SR), processing Glycyrrhiza uralensis (pGu), Fructus Viticis (FV), and Chuanxiong Rhizome (CR).	Qianghuoshengshi decoction (QHSS), composed of seven botanical drugs (APR, NRR, LRR, SR, pGu, FV, CR) in a 5:5:2.5:2.5:2.5:1.5:2.5 mass ratio, was prepared by refluxing with water (1:10, g/mL) twice for one hour each, after which the combined filtrates were concentrated, dried, and the resulting dry extract was dissolved in methanol/water for analysis.	(Guo et al., 2021)
Xiaoxuming decoction (XXMD)	twelve botanical drugs: Paeoniae Radix Alba, Chuanxiong Rhizoma, Saposhnikovia Radix, Stephaniae Tetrandrae Radix, Aconiti Lateralis Radix Praeparata, Glycyrrhizae Radix et Rhizoma, Cinnamomi Ramulus, Scutellariae Radix, Armeniacae Semen Amarum, Ephedrae Herba, Ginseng Radix et Rhizoma, Zingiberis Rhizoma Recens	Not reported	(Yang et al., 2019)

3 References

Cai, Z., He, M., Li, C., Qi, H., Bai, R., Yang, J., et al. (2023). Identification of chrysanthemum using hyperspectral imaging based on few-shot class incremental learning. *Computers and Electronics in Agriculture* 215, 108371. doi: 10.1016/j.compag.2023.108371

Chang, Y., Zhou, D., Tang, Y., Ou, S., and Wang, S. (2024). An improved deep learning network for image detection and its application in Dendrobii caulis decoction piece. *Sci Rep* 14, 13505. doi: 10.1038/s41598-024-63398-w

Chen, X., Yang, D., Huang, L., Li, M., Gao, J., Liu, C., et al. (2024). Comparison and identification of aroma components in 21 kinds of frankincense with variety and region based on the odor intensity characteristic spectrum constructed by HS–SPME–GC–MS combined with E-nose. *Food Research International* 195, 114942. doi: 10.1016/j.foodres.2024.114942

He, C., Shi, X., Lin, H., Li, Q., Xia, F., Shen, G., et al. (2024). The combination of HSI and NMR techniques with deep learning for identification of geographical origin and GI markers of Lycium barbarum L. *Food Chemistry* 461, 140903. doi: 10.1016/j.foodchem.2024.140903

He, S., Zhang, X., Lu, S., Zhu, T., Sun, G., and Sun, X. (2019). A Computational Toxicology Approach to Screen the Hepatotoxic Ingredients in Traditional Chinese Medicines: Polygonum multiflorum Thunb as a Case Study. *Biomolecules* 9, 577. doi: 10.3390/biom9100577

Husaini, A. M., Haq, S. A. U., Shabir, A., Wani, A. B., and Dedmari, M. A. (2022). The menace of saffron adulteration: Low-cost rapid identification of fake look-alike saffron using foldscope and machine learning technology. *Front Plant Sci* 13, 945291. doi: 10.3389/fpls.2022.945291

Ji, L., Zhang, Y., Li, Y., Song, L., Zhang, T., Du, C., et al. (2025). Concoctive principles of detoxification and retention of the main toxicity of Tripterygium wilfordii and its anti-inflammatory efficacy by concocting with the medicinal excipient Spatholobi Caulis juice. *Fitoterapia* 181, 106400. doi: 10.1016/j.fitote.2025.106400

Li, H., Gui, X., Wang, P., Yue, Y., Li, H., Fan, X., et al. (2024a). Research on rapid quality identification method of Panax notoginseng powder based on artificial intelligence sensory technology and multi-source information fusion technology. *Food Chemistry* 440, 138210. doi: 10.1016/j.foodchem.2023.138210

Li, M., Shi, Y., Zhang, J., Wan, X., Fang, J., Wu, Y., et al. (2023). Rapid evaluation of Ziziphi Spinosae Semen and its adulterants based on the combination of FT-NIR and multivariate algorithms. *Food Chemistry: X* 20, 101022. doi: 10.1016/j.fochx.2023.101022

Li, X., Yang, Y., Zhu, Y., Ben, A., and Qi, J. (2022a). A novel strategy for discriminating different cultivation and screening odor and taste flavor compounds in xinhui tangerine peel using E-nose, E-tongue, and chemometrics. *Food Chemistry* 384, 132519. doi: 10.1016/j.foodchem.2022.132519

Li, Y., Bi, Q., Wei, W., Yao, C., Zhang, J., and Guo, D. (2022b). Sequential decision fusion pipeline for the high-throughput species recognition of medicinal caterpillar fungus by using ATR-FTIR. *Microchemical Journal* 179, 107437. doi: 10.1016/j.microc.2022.107437

Li, Y., Zhao, W., Qian, M., Wen, Z., Bai, W., Zeng, X., et al. (2024b). Recent advances in the authentication (geographical origins, varieties and aging time) of tangerine peel (Citri reticulatae pericarpium): A review. *Food Chemistry* 442, 138531. doi: 10.1016/j.foodchem.2024.138531

Liu, J., Zhou, Y., Zhou, P., He, T., Liu, P., Wang, J., et al. (2024). Mechanistic insights into xanthomicrol as the active anti-HCC ingredient of Phytolacca acinosa Roxb.: A network pharmacology analysis and transcriptomics integrated experimental verification. *Journal of Ethnopharmacology* 333, 118467. doi: 10.1016/j.jep.2024.118467

Nile, S. H., Su, J., Wu, D., Wang, L., Hu, J., Sieniawska, E., et al. (2021). Fritillaria thunbergii miq. (zhe beimu): A review on its traditional uses, phytochemical profile and pharmacological properties. *Food Chem Toxicol* 153, 112289. doi: 10.1016/j.fct.2021.112289

Guo, J., Zhang, L., Shang, Y., Yang, X., Li, J., He, J., et al. (2021). A strategy for intelligent chemical profiling-guided precise quantitation of multi-components in traditional Chinese medicine formulae-QiangHuoShengShi decoction. *J. Chromatogr. A* 1649, 462178. doi: 10.1016/j.chroma.2021.462178

Yang, S., Shen, Y., Lu, W., Yang, Y., Wang, H., Li, L., et al. (2019). Evaluation and identification of the neuroprotective compounds of xiaoxuming decoction by machine learning: A novel mode to explore the combination rules in traditional Chinese medicine prescription. *Biomed Res Int* 2019, 6847685. doi: 10.1155/2019/6847685
